# A Unifying Review of Bioassay-Guided Fractionation, Effect-Directed Analysis and Related Techniques

**DOI:** 10.3390/s120709181

**Published:** 2012-07-04

**Authors:** Michael G. Weller

**Affiliations:** Division 1.5 Protein Analysis, BAM Federal Institute for Materials Research and Testing, Richard-Willstätter-Strasse 11, 12489 Berlin, Germany; E-Mail: michael.weller@bam.de; Tel.: +49-30-8104-1150; Fax: +49-30-8104-1157

**Keywords:** toxicity, drug discovery, natural products, environmental analysis, lead identification

## Abstract

The success of modern methods in analytical chemistry sometimes obscures the problem that the ever increasing amount of analytical data does not necessarily give more insight of practical relevance. As alternative approaches, toxicity- and bioactivity-based assays can deliver valuable information about biological effects of complex materials in humans, other species or even ecosystems. However, the observed effects often cannot be clearly assigned to specific chemical compounds. In these cases, the establishment of an unambiguous cause-effect relationship is not possible. Effect-directed analysis tries to interconnect instrumental analytical techniques with a biological/biochemical entity, which identifies or isolates substances of biological relevance. Successful application has been demonstrated in many fields, either as proof-of-principle studies or even for complex samples. This review discusses the different approaches, advantages and limitations and finally shows some practical examples. The broad emergence of effect-directed analytical concepts might lead to a true paradigm shift in analytical chemistry, away from ever growing lists of chemical compounds. The connection of biological effects with the identification and quantification of molecular entities leads to relevant answers to many real life questions.

## 1. Introduction

The development of more and more powerful analytical methods is an ongoing process and lays the foundation of many scientific, technological and medical advances. One example is the fierce advancement of the field of mass spectrometry, which had been considered as largely mature for some time. However, the analytical information explosion caused by these technological improvements may finally lead to an information overload [1]. To pick out the relevant results out of the vast amount of data is by no means a trivial task. The emergence of the new scientific field of bioinformatics is one way to try to tame this mass of information. Unfortunately, the purely mathematical treatment of experimental data cannot replace lacking knowledge or understanding in general. One of the ways out of this problematic situation is to abandon the purely chemical and mathematical perspective and to turn to the concept of biomolecular interaction, which is one of the most influential ideas in pharmacology and toxicology. Paul Ehrlich (1854–1915) put it shortly in his fundamental principle *Corpora non agunt nisi fixata* (Drugs do not act unless they are bound) [2,3]. Or to put it the other way round: only compounds binding to biological targets are of interest. Based on this rationale, it becomes evident that a “biomolecular interaction step” should be introduced into analytical methods to achieve bio-selectivity and to focus on relevant compounds. How to achieve this is the main topic of this review.

## 2. General Concept

The first prerequisite for the application of the concepts discussed here is the necessity to analyze a complex sample, which cannot be assessed completely by conventional means. In most systems, some sort of separation is applied to reduce the complexity of the fractions. Sometimes, even several separation steps might be required. The second and most characteristic part is the biological or biochemical entity, which can be as small as a peptide or complex such as a living cell or even an animal, e.g., a fish. Finally, identification, structural analysis and quantification steps can be applied to assign chemical structure and a physical amount to the respective compound(s). To confirm the results, validation by comparison with a pure compound should be performed.

## 3. Nomenclature

The concepts described in this review most likely have been developed independently in several fields. This complex history led to an extraordinary heterogeneous nomenclature. In Table 1 some of the most important designations are listed with the number of references in ISI Web of Science. As the sum of the references already reaches several thousands in 2012, it is obvious that only a more or less arbitrary selection can be cited or discussed here.

Not covered by this review is the analogous concept of bioactivity-guided or biology-oriented synthesis, which was discussed recently by other authors [4–6]. This approach uses bioactivity information to design new scaffold-based (focused) libraries and to explore the “chemical space” around validated structure elements with confirmed activity.

Frequently used terms are bioassay- or (bio)activity-guided fractionation, biochemical detection, and bioactivity screening. Most of them are mainly connected with drug discovery. Others, such as effect-directed analysis or bioresponse-linked instrumental analysis are more rooted in the environmental sciences. In contrast to a common practice, I do not want to suggest a standardized nomenclature. It seems to be sufficient to improve the awareness that many quasi-synonyms exist and should be considered e.g., in literature searches or feasibility studies.

Obviously, the most characteristic point is the biological or biochemical part, which has to select or indicate compounds, which interact with the biological/biochemical entity. It gives “biological relevance” to the result. Usually, the binding to an artificially generated antibody, aptamer or fully synthetic binder is not of interest in this context, because in most cases, they have no special biological function or meaning.

Another important part is the separation step. This can be a very simple fractionation into a few fractions or a sophisticated high-performance chromatography or electrophoresis. The aim is to avoid the simultaneous presence of different compounds in the bioaffinity interaction step. The examination of the effect or toxicity of mixtures [63,64] is a very complex and highly debated field. Synergistic, antagonistic or additive toxicity can occur, which is extremely difficult to predict [65,66]. To avoid these problems, a base-line separation of all chemical compounds should be intended in effect-related analysis. The final part is a detection step, which is necessary to generate a signal (e.g., UV absorbance). This can be also combined with all kinds of structural analysis, such as mass spectrometry, NMR, Raman or IR. In addition, a quantitation of the respective compound(s) might be included. In many systems the correlation between a biointeraction signal and a physicochemical signal is important to assign a compound to an “effect”. This assignment usually is performed by examination of the corresponding retention times or R_f_ values.

## 4. Reviews and Important Papers of the Topic

Only a few reviews have been published in this field, also underlining the notion that the generalizability and value of the concept is not sufficiently acknowledged. A review about the technical aspects of bioassay-guided fractionation has not been presented to my knowledge, although the methods seem to have been in use for such a long time that the identification of the “true” inventors is difficult. Nearly all papers are focused on the drug, the environmental sample or the natural product itself and less on the analytical process.

In 1974 Commoner *et al.* presented a study about carcinogens in rat urine [67]. The researchers separated the urine into a few fractions to examine carcinogenic metabolites. A similar study has been published by Durston and Ames [68]. The *Symposium on Application of Short-Term Bioassays in the Fractionation and Analysis of Complex Environmental Mixtures* held at Williamsburg (VA, USA), 21–23 February 1978, led to a comprehensive *Proceedings* volume [69] describing the diversity of applications, which had been explored in such a short time after the ground-breaking publications of the group of Bruce Ames.

A more elaborated thin-layer chromatographic fractionation of coal tar and the subsequent examination of mutagenicity was published by Wilson *et al.*, in 1980 [70] and Bjorseth *et al.*, in 1982 [71]. They also used the highly popular Ames-Test based on the mutation of strains of the bacterium *Salmonella typhimurium* [72–76].

Schuetzle and Lewtas [36] published an influential paper in 1986. They examined the question of which fractions or compounds of diesel exhaust are responsible for its mutagenic effects. Related work was performed by Arey *et al.* [77], who presented “HPLC mutagrams” also based on the popular Ames test. A review about the topic was published by Marvin and Hewitt in the year 2007 [78]. The area of “effect-directed analysis” and “toxicity identification evaluation” was briefly reviewed in a recent paper of Ballesteros-Gómez and Rubio [79] under the section “Integrated Chemical and Biomonitoring Strategies”. In 2005 a review covering the effects-directed investigation of effluents was published by Hewitt and Marvin [58]. In the year 2003, the concept of “effect-directed analysis” was described and discussed in detail by Brack [32]. In this publication, he gave an overview of applications, mainly in environmental analysis. In 2008 another review was published by the group focusing on the confirmation problem in “effect-directed analysis” [33]. 2001, Reemtsma published a review about applications and prospects in wastewater analysis [80]. “Bioautography detection in thin-layer chromatography” is the title of a review of Choma and Grzelak [81]. Recently, a review about “Hyphenations in planar chromatography” was published by Morlock and Schwack [82]. The application of effect-directed analysis (EDA) in combination with thin-layer chromatography (TLC) covers a significant part of the paper. A similar topic was covered by a paper and a doctoral thesis of Weins [83,84]. A review about applications in phytochemistry was published by Marston [85].

In the field of drug discovery, conceptual reviews are also rare. An instructive review was published Potterat and Hamburger [86]. The challenges of drug discovery (e.g., unrealistically high hit rates in high-throughput screening, HTS) are discussed and the limitations of the different approaches are critically reviewed. Recently, Kreiss *et al.* published an interesting paper showing a combination of a whole-cell bioluminescent reporter gene assay with TLC for the examination of extracts of natural compounds, followed by HPLC/MS and HPLC/NMR for dereplication (elimination of compounds already known) and structure elucidation [87].

A very active group in the field of the development of novel analytical approaches—particularly for drug development—is the group of Irth in Amsterdam [19,21,22,36,88,89]. One of their research interests lies in the hyphenation of liquid chromatographic methods with “biochemical detectors”, which means systems being able to detect biochemical interactions online.

Gas chromatography with olfactometric detection (GC-O) is a special, but very important variant of effect-related analysis. The examination of food, water, indoor air or fragrances was shown in many papers. An interesting review was published by Muñoz *et al.*, discussing the odorous emissions of waste water [90]. Electronic noses are also examined in this context. However, these are not covered by this review, since they lack the similarity to a biological response. Biosensing based on isolated olfactory receptors [91,92], which might be able to imitate the respective biological systems, seems to come into reach. The older variant of gas chromatography with electroantennographic detection (GC-EAD) has been a powerful tool for the examination of insect pheromones since many years [26,93–96]. A nice overview was given in [97].

## 5. Separation

### 5.1. Liquid Chromatography

Liquid chromatography, including low-pressure LC, HPLC [98–100], UPLC, counter current chromatography (CCC, [101–105]) and other variants are one of the most obvious methods for an off-line or online separation of complex mixtures. A big advantage is the ease to realize hyphenation to other liquid-based systems, such as autosamplers, detectors and fractionators, many of which are commercially available. For the clean-up of complex natural extracts, the use of CCC is very popular due to its robustness. However, its separation power is relative limited and up to now, only very few applications in the field of effect-directed analysis were reported [106,107]. The disadvantages of liquid chromatography are the use of organic solvents and other additives, which are rarely compatible with bio- or biochemical assays. Particularly difficult are gradient-based separations, in which the solvent composition is changing heavily. In this case, an evaporation step is often the best way to eliminate interferences. However, volatile compounds might be lost. Another problem are fast separations, which often do not match the time frame needed for the bioassay. Therefore, not many systems have been presented, in which a true online hyphenation could be shown, e.g., [18,31,36,44].

### 5.2. Gel Chromatography

Gel chromatographic separation steps (or alternatively field-flow fractionation FFF, [108]) are mainly used in biochemical fields, where proteins and other biopolymers have to be treated. Unfortunately, the separation efficiency is not very high and a more or less pronounced dilution effect has to be accepted. On the other hand, the mainly aqueous solvent/buffer often used in an isocratic mode is well suited to be coupled to any biological or biochemical assay or step. Unfortunately, only very few applications have been reported yet [53].

### 5.3. Thin-Layer Chromatography (TLC)

Perhaps surprisingly, one of the most popular separation techniques for effect-related applications is thin-layer chromatography (TLC). Most of the systems take advantage of the good availability and ease of application of bioluminescent bacteria (formerly *Vibrio fischeri*, now *Aliivibrio fischeri*), which are routinely used for general toxicity tests. However, other organisms, such as the yeast *Candida albicans*, have been used for a similar purpose [14]. A group at Bayer (Leverkusen, Germany), was one of the first to combine a thin-layer separation with bioluminescent bacteria [35]. Particularly, the variant called AMD (Automated Multiple Development) seems to be useful [98]. One reason might be the better separation, another complete removal of residual solvent, which might inhibit the bacteria. Recently, a paper was presented, in which the application of the luminescent bacteria was not performed by dipping in a suspension, but by rolling [109]. This reduces blurring and tailing of polar compounds and hence, improves the performance of the technique.

### 5.4. Gas Chromatography (GC)

For volatile compounds, the separation by gas chromatography is unrivaled, due to its extreme efficiency and excellent detection sensitivity in combination of mass spectrometry. Another advantage is the availability of huge data libraries, which allows the tentative identification of compounds in minutes. Unfortunately, not many biochemical systems can be used in the gas phase. However, some applications have gained significant importance, such as the examination of flavors, e.g., in food or fragrance analysis. Here, the human nose is used as a “sniff detector”. The method is known as GC-olfactometry [11], often combined with a mass spectrometer. By determination of dilution factors, this approach even can be used in a (semi-)quantitative way [110]. Typical applications are the examination of key odorants of wine [111], cheese [112] or olive oil [113]. A much more exotic approach is gas chromatography with electroantennographic detection (GC-EAD) for the examination of insect pheromones [94,96,114]. One group combined GC × GC/TOFMS with GC-EAD to examine pheromones not detectable by FID [115].

### 5.5. Affinity Enrichment

Affinity chromatographic methods are very important in bioanalysis [116]. However, by far most of them are based on artificially generated antibodies or synthetic ligands and usually do not generate a biological information. Only very few are based e.g., on human receptors, which can be used to examine the binding of molecules of interest (e.g., inhibitors). It was tried to identify endocrine disruptors in environmental samples by a selection step based on a column loaded with the respective receptor. In a paper of Seifert *et al.*, the concept was described in some detail, however, without experimental results [58]. Recently, a doctoral thesis [117] published at RWTH Aachen, Germany, described a similar attempt to identify unknown estrogenic compounds in sewage water. The affinity enrichment led to an improvement of the signal-to-noise ratios in the LC-MS/MS chromatograms of the known estrogens; one new compound could be tentatively identified. A similar approach was tried with magnetic beads coated with human estrogen receptors [118]. The model system was demonstrated with an eight-compound mixture–however, real samples were not analyzed, yet. A food analytical application of an affinity column with immobilized estrogen receptors was shown by Riu *et al.* [119].

## 6. Collection of the Separated Fractions

### 6.1. Manual Fractionation

One of the simplest approaches is the manual fractionation of a raw sample by chromatography, electrophoresis or other separation technique. Subsequent testing of the fractions in a biological system, e.g., the water flea, *Daphnia magna*, completes already a bioassay-guided fractionation system. Usually, the fractions are collected in a time-controlled way. The other option is to collect only some major peaks in a chromatogram. This limits the time and effort required for the “bioassay” considerably. Here, even tests with living animals might be feasible. However, it is possible that the most interesting compounds are lost in the fractionation step, since the peak intensity (absorbance in case of UV detection) was too low. Usually, the separation system is not directly coupled to the biological/biochemical unit. In this case, a manual transfer is necessary. However, this approach has obvious advantages: The separation and the testing are temporally and spatially decoupled. Enrichment, evaporation, pH adjustment, dilution or repeated measurements under different conditions can be performed easily. Manual or automated fractionation is also well-known from the purification of enzymes, which could be considered to be an “enzyme activity-directed fractionation”. Here only relatively large fractions are cut, which still contain a large number of different compounds. The fraction of the highest activity is taken and subjected to a second, different separation step. This is repeated (sometimes many steps are required), until the specific activity (activity per weight) becomes more or less constant, which means that the enzyme has been “purified to homogeneity”. However, this still does not mean 100% purity; sometimes simply no other method for a further separation is available. Although these approaches might seem outdated, most likely they are one of the most popular and common ones and are extremely valuable in the respective fields.

### 6.2. Automated Fractionation

**Figure 1.**
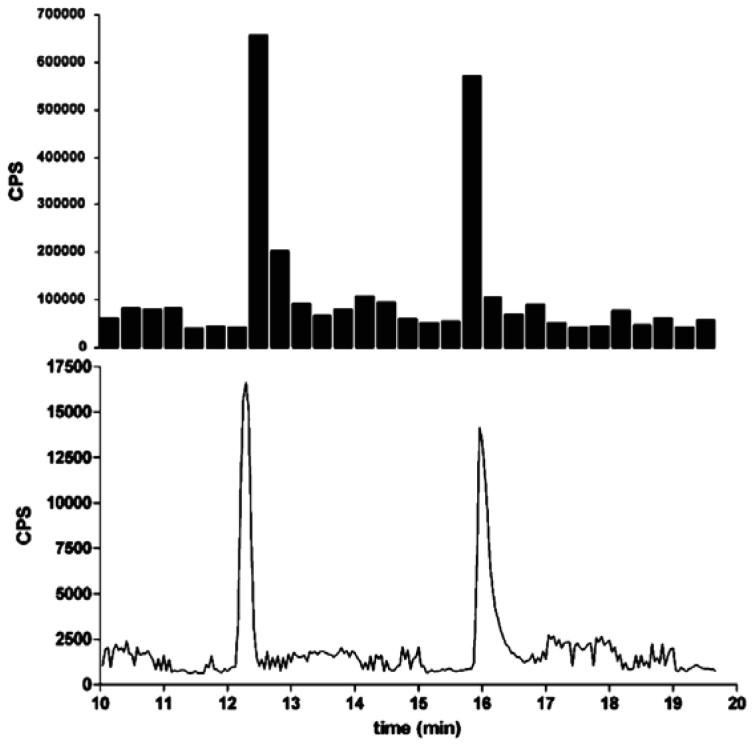
Comparison of parts of a fractionation in 96-well (20 s per fraction) and 1,536-well (2.5 s per fraction) microtitration plates (MTP). CPS counts per second. Reprinted with permission from [120]. Copyright (2009) American Chemical Society.

A minor improvement is the introduction of automated fractionation devices, which are able to cut fractions in the range of milliliters down to nanoliters in glass vials, reaction tubes, 96-well and 384-well microtitration plates and on glass slides with different coatings. Even a 1,536-well screening assay was demonstrated [120]. The advantage in resolution can be seen in Figure 1. A time-controlled fractionation is most frequently used. Nevertheless, some effort might be needed, to get the whole chromatographic run stored in the vessels. In case of an incompatibility of the volume streams, splitting might be necessary, although a large fraction of the sample will be lost. In addition, the fraction size has to be adjusted to the separation efficiency. Also, the compatibility of the analytes and solvents with the material of the vessel or microtitration plate has to be considered. Some compounds tend to adsorb at polymer or glass surfaces or show only minimal solubility in aqueous media. A special case is the continuous deposition of the chromatographic stream on a surface, for example in a meandering format. Usually the solvent evaporates immediately and the separated compounds are stored on the surface in a more or less dry form. This is particularly popular on MALDI-TOF-MS targets, where the continuous addition of the MALDI-matrix can be included. However, a coupling to a bioassay is not straightforward.

### 6.3. In-Plane Fractionation

A special form fractionation is the use of a thin layer chromatographic plate or electrophoretic gel. Here, the silica layer or the gel itself is the storage location of the separated compounds. The bioassay either will be performed in the pores or a kind of blotting technique has to be applied to transfer the compounds from the plate to a more suitable environment. Alternatively, some bands or dots can be cut, extracted and transferred to a bioassay system.

### 6.4. Fractionation by Liquid Plugs or Gas Bubbles

In a closed system, the fractionation by bubbles or immiscible liquid plugs is an option to reduce backmixing and to make post-column bioassays technically more feasible [31]. The problem to be solved is the incompatibility of the time-scale of the separation, which would require detection in the range of milliseconds and of the bioassay, which needs reaction times of at least some seconds or even minutes. Good post-column reactions have to be very fast, which can be achieved either by the use of high reagent concentrations and/or high temperatures. Both approaches are obviously not suitable for biochemical reactions. Therefore, a kind of temporal decoupling has to be achieved between the separation and the detection step. From a strict point of view, one could object that this is not a real online hyphenation, because there is no instant detection, but a significant delay involved, dependent on the required time for the biochemical assay. However, for nearly all applications, this delay—although of significant duration—is not of major importance.

## 7. Bioaffinity Components

### 7.1. Living Organisms

The use of living animals is one of the most obvious approaches for toxicity-related questions. Rodents, fish/fish eggs and daphnia are frequently used for this purpose. However, relatively large animals are not very practical for the application in effect-related analysis. Microorganisms, in contrast, are well-suited for effect-related questions. However, their exposure periods are often long, in the range of hours or even days [121]. As already mentioned, the Ames test, based on the mutation of special strains of *Salmonella typhimurium*, was frequently used for many experiments dealing with samples containing mutagenic compounds, such as polycyclic aromatic hydrocarbons. Another very popular system is based on the inhibition of bioluminescent bacteria (formerly *Vibrio fischeri*, now *Aliivibrio fischeri*), which generates a parameter of general toxicity, particularly used in water analysis (e.g., EN ISO 11348). Parvez *et al.*, concluded that luminescence assays based on *V. fischeri* are very useful for the estimation of acute toxicity of a broad spectrum of chemicals [122], not only in water samples. Bacterial bioluminescence is well suited for the use in combination with thin-layer chromatographic plates. Inhibitory compounds are identified after the coating of the plates with the bacteria suspension as dark spots. *Vibrio/Aliivibrio fischeri* can be easily obtained in a freeze dried form and can be considered as non-hazardous. Although organisms are good models to test samples or fractions for toxicity, they have disadvantages of poor stability and the necessity of some cultivation in appropriate media. Particularly organic solvents often interfere heavily. An innovative approach is the application of the chemically activated luciferase gene expression (CALUX) bioassay [15,123] and similar systems [124]. It was used for the examination of atmospheric deposition [125], food and feed samples for contaminations of polychlorinated dioxins, furans, biphenyls and for the study of estrogenic compounds [126], as well. Another option is the use of the yeast estrogen screen (YES) system [127–131]. Routine examination of animal feed for estrogens was performed based on a LC/YES-bioassay/QTOFMS setup [132]. For the test on herbicides, the use of algae is useful. Brack [32] and others emphasized that the application of only one test species is not sufficient for a comprehensive examination of toxic hazards. In this case, the setup of a biotest battery of complementary species is highly recommended [133]. Unfortunately, most of the organism-based assays—recombinant or not—are rather slow, which causes some problems in online hyphenations. For *in vitro* assays, evaporation of the solvent is highly desirable to avoid cytotoxic effects of the mobile phase. However, the solid film that stays after such an evaporation step may inhibit cell adhesion in the well plate. Alternatively, to reduce the solvent content, the fractions might be diluted in a suitable medium, which unfortunately also leads to a reduction of the concentration of the active compounds.

### 7.2. Organs or Organelles

Electroantennographic detection is based on the use of fresh insect (or other arthropod) antennae. The changes of the electric potential are recorded and show the reaction of the organ on compounds in the gas phase or in water. In combination with a gas chromatographic system (GC-EAD) a popular setup is formed. For example, bees, locusts, cockroaches and lobsters are used for this purpose. Organelles, such as chloroplasts, might be also used for effect-related analysis [134], e.g., for the identification of photosynthetic inhibitors, which might be useful for the development or detection of herbicides.

### 7.3. Enzymes

Enzymes are excellent tools for effect-related analytical systems. They are often well-studied pharmacological or toxicological targets and can be obtained either by extraction/isolation from natural sources or produced via recombinant technologies e.g., in *E. coli*, yeasts, CHO cells or other expression systems. The primary property of an enzyme—the catalytic activity—is particularly useful for our analytical purposes. Often chromogenic or fluorogenic substrates are available, which allow the continuous measurement of the enzyme activity, which can be inhibited by different classes of compounds. These inhibitors can be either potential pharmacological leads or potential toxicants. In most cases, enzyme inhibition is a fast reaction in relation to toxicity on organisms. Nevertheless, they are relatively slow form post-column application, which makes it often necessary to decouple or delay the detection step.

### 7.4. Affinity Binders

Most antibodies cannot be considered to be biochemical components of effect-related systems. Therefore, most general affinity or immunoaffinity systems [116] are out of the scope of this review. The same applies mostly to DNA, RNA, oligonucleotides, aptamers and so on. In contrast, (human) receptors are extremely valuable for this purpose. Unfortunately, the use of a functional receptor system is quite difficult outside of a living organism. Therefore, nearly all receptor assays are used more or less similar to a simple (antibody-based) binding assay. Hence, it is not possible to differentiate agonistic and antagonistic effects in this detection mode. It has to be kept in mind that receptor binding in these tests has not the same meaning as the occurrence of a biological effect. The first type of such assays was based on radioactively labeled hormones, which allows the use of quite crude receptor preparations and in addition, is quite sensitive. Today, assays based on fluorescently labeled compounds are more popular and well suited for the introduction in an effect-related analytical system. However, the availability of (human) receptors is limited. In addition, their stability is often poor, compared to antibodies or other general biochemical reagents. An online system for the investigation of biosynthetic estrogenic compounds was presented by de Vlieger *et al.* [135]. In this paper, the parallel use of two different receptors was shown. In combination with off-line NMR several unknown products could be identified.

## 8. Detection

### 8.1. Instrumental Detectors

The detection step in an effect-related analysis system has a twofold purpose. First of all, it shows the presence of a compound, for example in a chromatographic elution. Here, a (relatively) non-specific detector is most suitable, such as UV, refractive index (RI), evaporative light scattering (ELSD) or charged aerosol detectors (CAD). A semi-quantitative or a quantitative determination is often desirable. The second purpose, which often might be the primary, is the elucidation of the structure of an unknown compound. Here, mass-spectrometry (particularly based on ion traps) is dominant, since they deliver plenty of structural information with only a minute amount of substance required. The most valuable approach seems to be GC/MS in combination with a mass spectral library (e.g., from NIST), which facilitates the (tentative) identification of an unknown compound in a complex sample. However, extreme care is needed in this assignment step. Only after a validation step, e.g., by comparison with a reference compound, the compound can be considered to be identified. Very helpful are GC retention indices, which are available as large libraries, too. Another approach is the use of high-resolution MS, which allows the assignment of an elemental formula instantly. However, the most powerful method for chemical structural analysis is most likely NMR (nuclear magnetic resonance). Unfortunately, the required substance amounts are much higher, making this approach often unfeasible in our context. In addition, the accumulation time might be in the range of hours, which limits the use in on-line setups.

### 8.2. Biodetectors

**Figure 2.**
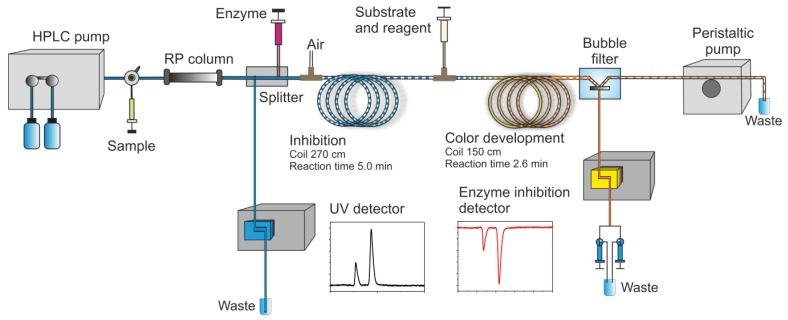
HPLC with Segmented Flow Enzyme Inhibition (HPLC-SFEID). Drawing courtesy of Susanne Fabel [31], modified.

**Figure 3.**
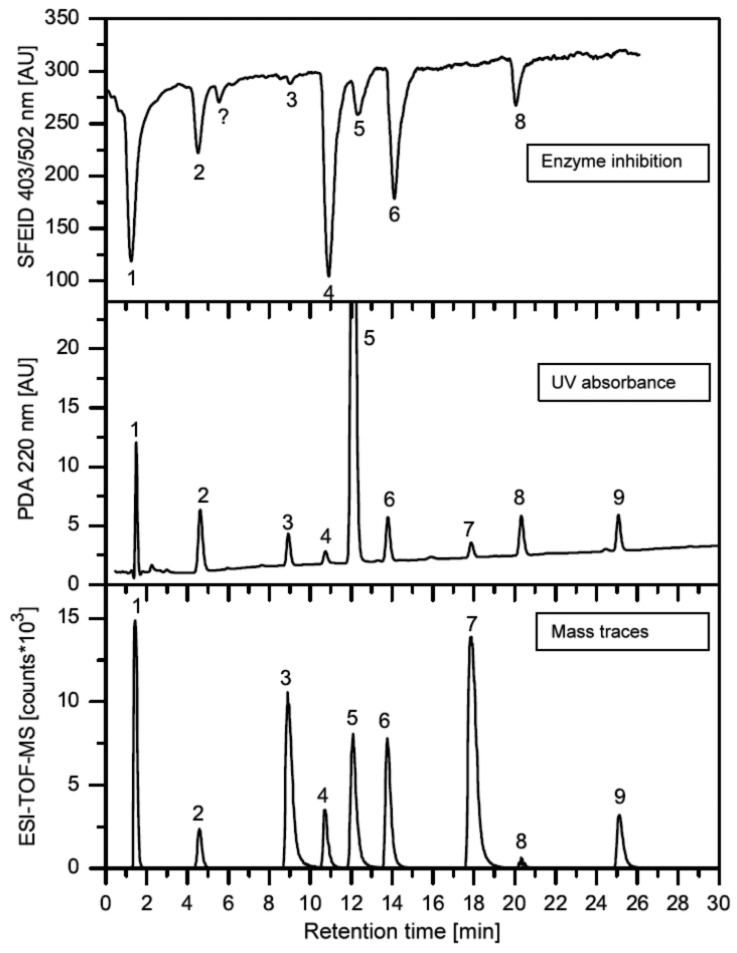
Application of a HPLC-SFEID system for the examination of acetylcholine esterase inhibitors: (1) galanthamine; (2) methomyl; (3) aldicarb; (4) dichlorvos; (5) carbaryl; (6) paraoxon; (7) malathion; (8) parathion; (9) chlorpyrifos. Please note the very different responses in UV absorbance, mass spectrometry and enzyme inhibition. Figure courtesy of Susanne Fabel [137].

**Figure 4.**
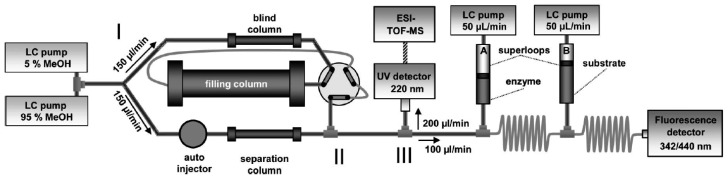
Biochemical detector with a countergradient parking system to reduce solvent inhibition effects [88]. Reprinted with permission from [88]. Copyright (2008) American Chemical Society.

In this section, some exemplary systems with bio(chemical) detetection are shown. Fabel *et al.* [31] have developed a setup for the analysis of acetylcholinesterase inhibitors (AChEI), such as toxic insecticides from the organophosphate and the carbamate group. This approach (Figures 2 and 3) was termed HPLC with Segmented Flow Enzyme Inhibition (HPLC-SFEID). In a first step, the sample is separated by a conventional reversed-phase HPLC. After the column the mobile phase is splitted into two branches, one flowing to the UV detector and ESI-TOF-MS (not shown) and the other to the enzyme inhibition detector. In the latter, the eluent of the HPLC is mixed with a buffered enzyme (AChEI) solution. Directly after the mixing-T, air bubbles are introduced. The gas segmentation leads to an extremely small peak broadening and makes long inhibition or other incubation times possible. After a first incubation (inhibition) step, a suitable colorimetric enzyme substrate was added by another syringe pump. Most detectors are disturbed by air, and therefore, a bubble filter was needed at the end of the system. The photodiode array (UV detector) was used for substance detection and confirmation, the ESI-TOF-MS for (tentative) identification. Although not shown, chemical weapons (e.g., Sarin, Soman, Tabun, VX and others) also might be detected and identified by this system based on acetylcholinesterase inhibition. Carol-Visser *et al.*, designed an on-line system for the detection of Sarin and sulfur mustard by pepsin digestion followed by LC-MS focused on specific peptides, originating from the respective chemical reaction by the toxic compound [136]. To avoid unwanted effects of the solvent gradient, a countergradient system was presented by Schebb *et al.* [88]. The setup is shown in Figure 4. In a countergradient, a second solvent gradient is produced this way that the mixing of the two gradients after the separation column leads to a constant concentration of organic solvent. This concept addresses the problem of highly drifting baselines in online inhibition detectors without the need for a solvent evaporation step.

**Figure 5.**
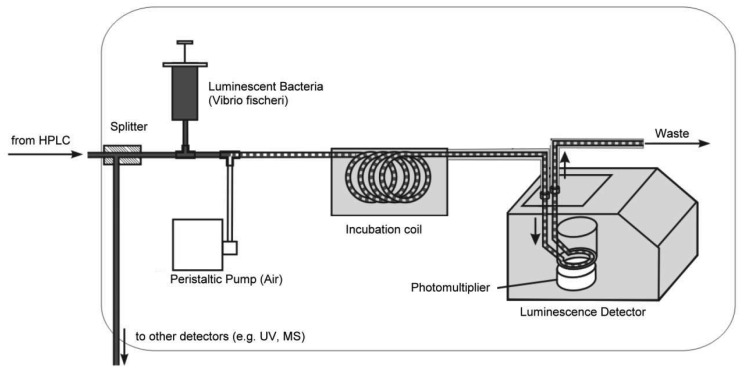
Setup of an online-bioluminescence detector with luminescent bacteria. Figure courtesy of Philipp Stolper [23,139], modified. In this system, a removal of the air bubbles in front of the detector is not necessary. However, leakage of stray light through the tubing needs to be carefully avoided.

**Figure 6.**
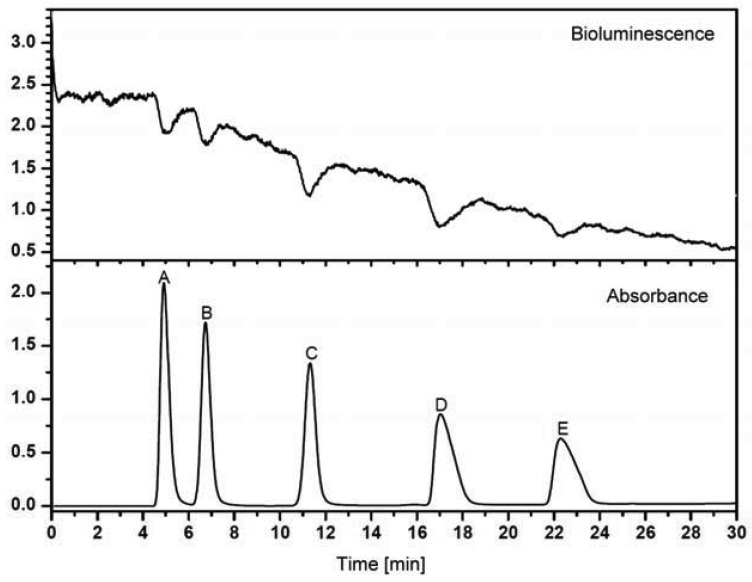
Effect-related analysis with Bioluminescence Inhibition Detector (BID, upper trace) and Photodiode Array Detector (PDA, lower trace). (A): 2,4-dinitrophenol (200 mg/L); (B): 4-fluoro-2-nitrophenol (300 mg/L); (C): 2-methyl-4,6-dinitrophenol (200 mg/L); (D): 3,4-dichlorphenol (300 mg/L); (E): 3,5-dichlorphenol (300 mg/L). Please note the decreasing baseline of the bioluminescence, most likely caused by the water/methanol gradient. In addition, the relative poor sensitivity has to be considered, which might be due to the insufficient incubation time in the online system (see “Challenges” below). Figure courtesy of Philipp Stolper, modified [139].

Recently, a microfluidic system for the detection of compounds interacting with an acetylcholine binding protein was shown [138]. A nano-LC device was coupled to a miniaturized biochemical detector (BCD) to reduce the consumption of expensive reagents, which is a critical point for the applications of such a detection system. But not only enzymes or receptors are suitable biocomponents for effect-related approaches. Microorganisms are excellent means to get some toxicology-related data. Particularly, luminescent bacteria are very popular in this context. Stolper *et al.*, have designed a flow-through luminometer on base of a commercial device (Figures 5 and 6, [23,139]). In combination with a gas-segmented fluidic system, an online bioluminescence inhibition detector was constructed. Unfortunately, the long-term stability of living microorganisms is still a problem [140,141].

## 9. Current and Potential Applications

### 9.1. Toxicology

Toxicological studies are one of the most interesting applications in this context. Without effect-related analysis, a clear assignment of a toxic action of a real sample to a chemical compound is hardly possible. Even if suspicious “pure compounds” are directly subjected to toxicity tests, purity or identity questions may arise. Synthetic byproducts or degradation products nearly always contaminate the “pure compound” with unknown, unexpected or unwanted active derivatives. Using effect-related analytical systems can avoid or at least reduce such doubts and troubles.

### 9.2. Natural Compound Screening

This seems to be one of the most frequent applications. Except for studies of pure basic research, nearly all examinations of extracts of natural materials are based on an interest for active ingredients. Unfortunately, natural extracts are often extremely complex and contain many unknown compounds. In this situation, the use of an effect-related analytical approach is a real relief [7]. When an active fraction or even compound is isolated, the identification or structural analysis is often quite straightforward. However, in many cases, this last step is hampered by a lack of sample, which might encourage the extraction of a larger amount of raw material. Similar applications can be imagined in general drug discovery and the decoding of synthetic libraries. A difficult issue is the dereplication of known (or non-specifically inhibiting) compounds, which are of no special interest in lead identification or drug discovery projects. A general approach of dereplication was shown e.g., with mycotoxins and fungal metabolites [142] and for a HIV inhibitor screening [143].

### 9.3. Environmental Analysis

One of the oldest applications of effect-related analysis, particularly for the examination of mutagenic compounds in aerosols or contaminations in sediments [144], is environmental analysis. However, also the examination of sewage water is becoming more and more interesting. In many cases, the identification or monitoring of endocrine disruptors is of upmost importance [145]. Unfortunately, the examination of surface or ground water is difficult, due to the extremely low concentrations of the respective contaminants, which are usually several orders of magnitude below any toxic or inhibitory level, which is obviously the prerequisite of any effect-related analysis. To overcome this concentration problem, some kind of enrichment can be applied (e.g., by solid-phase extraction SPE). The well-proven HPTLC (high-performance thin layer chromatography)/*Vibrio fischeri* combination was used for water analysis after SPE [146]. This step may lead to a selective enrichment, which removes most of the interfering matrix compounds, but on the other hand, may lead to losses of active compounds. If non-selective enrichments are used, the concentration of matrix compounds may lead to non-specific inhibition effects or losses of active compounds by adsorption or complexation, often observed with waters rich of humic and fulvic acids. In extreme cases, even non-extractable or bound residues might be formed [147,148]. Some authors presented setups for water analysis, which are technically similar to concepts of effect-related analysis [20,149]. However, since they use antibodies as affinity binders, they do not meet all the criteria, which we demand for such a system. HRGC-MS olfactometry has been successfully applied for the identification of a medicinal off-flavor in a mineral water [150]. The trace compounds 2-iodophenol and particularly 2-iodo-4-methylphenol could be assigned to be responsible for this malodor.

### 9.4. Food Analysis

Similar to drinking water, food is one of the materials, which comes into the closest contact with the human body. Therefore, effect-related analysis of such products may be particularly relevant. Some concepts based on thin-layer chromatography have been presented by Morlock and Schwack [151]. The same group also showed an innovative multienzyme inhibition system for the analysis of toxic insecticides in water or juice [152]. Another study examined natural aryl hydrocarbon-receptor agonists in marmalade [48]. Also, olfactory studies of food may be performed via the well-established gas chromatography-olfactometry method. However, not only smell, but also taste can be used in an effect-related approach, e.g., for the examination of mushrooms [153]. Systematic food control for toxicity by effect-related analysis seems not to be established, yet. However, this might be a new dimension of food control to achieve a higher level of safety, even when completely unknown contaminants are involved. One example was shown for the examination of a complex mixture of patulin-glutathion adducts [89], which are relevant for contaminated products from apples and other fruits. Another question is the identification of nutraceuticals or grading of food. Compounds of endocrine activity are also an issue in food analysis, and have been examined with affinity enrichment techniques based on immobilized estrogen receptors [119] or with a YES assay [131]. Value determining compounds (e.g., antioxidants) can be identified [154,155] and quantified with an effect-related approach. Finally, the screening of veterinary drugs in food [156] is an important potential field for the application of effect-related analysis.

### 9.5. Doping Analysis

*Doping analysis* seems to be a very urgent issue of analytical research [157]. Today, nearly all methods in doping control are based on classical instrumental tools (e.g., GC-MS, LC-MS/MS) or screening tests (immunoassays) [158]. The regular advent of novel compounds, which have been intentionally developed to fool analytical chemists and surveillance authorities, show the potential of effect-related approaches, which are guided by effects rather by masses or other physicochemical characteristics. Only very few papers have been published in this field, covering topics like the detection of illegal steroids [7]. The very illuminating case of the first detection of the designer steroid tetrahydrogestrinone (THG) shows that unknown compounds can remain hidden for quite a long time, when only standard analytical techniques are used for doping analysis [159–161].

### 9.6. Forensic Toxicology

Most forensic approaches are based on targeted analytical techniques, such as GC-MS in conjunction with large databases, containing many of the relevant, mainly toxic compounds. However, similar to the questions in doping analysis, unknown or rarely used active compounds may lead to a completely wrong result or interpretation of an analysis. Novel toxicants are often found only by the use of ancillary information, which is only available by chance. On the long run, the use of effect-related analytical techniques might completely change the way such analyses are performed. The application of biosensors have been shown already for the examination of stomach content [162], however not in an effect-related setup.

### 9.7. Pheromone Studies

The investigation of the behavior of insects often comprises the analysis of pheromones. These studies strongly profit from the application of effect-related approaches, which are a good way to discover novel pheromones [95,163–165], useful for basic research and insect control. One of the first papers on the antennal receptors of the silk moth was published by Schneider in the year 1957 [166].

## 10. Challenges

Effect-related analysis generates valuable results in many different application fields. However, the technology is not really widespread compared to conventional analytical techniques. Some significant limitations and challenges may be the reason for this slow proliferation. These—and perhaps even more—have to be considered, when the introduction of such a system is planned:

- Different time frames of separation/biotest/detection/analysis
- Different concentration ranges of separation/biotest/detection/analysis
- Sensitivity not sufficient for toxicity determination
- Sensitivity not sufficient for structural analysis, such as NMR
- Enrichment often necessary (e.g., SPE)
- Biotest/toxicity test too complex to be automated
- Poor availability of biocomponents, such as receptors
- Lack of stability of biocomponents
- Difficulties in miniaturization
- High costs of complex systems and reagents
- Lack of standardization, poor acceptance
- Extensive development needed - simple setups are rare
- Interdisciplinary field, broad knowledge needed
- Poor interaction between non-related application fields

For the selection of the right approach these questions have to be answered at least for the specific system. This can increase the value of the concept and probability for success considerably. Today, effect-related analysis cannot be considered to be an off-the-shelf technology.

## 11. Toxicity Models *vs.* Established Toxicity Tests

Most common toxicity tests, such as the determination of the LD_50_ in rats or most fish tests are hardly applicable for an effect-related analytical approach. The effect may occur too slowly (e.g., in 14 days) or too many larger animals might be necessary to test a highly fractionated sample. In this respect, at least partially reversible systems (e.g., living organs) or microorganisms (such as bacteria) are preferable. However, these simple systems are relatively far away from an elaborated toxicity investigation. This should be always taken into account, when such results are to be interpreted. Recombinant organisms might be useful, but their effect cascades are even more “artificial” and the results have to be discussed with great care and need additional validation. Therefore, most effect-related analytical systems are primarily screening tools to identify novel compounds with relevant properties in a complex sample. Ideally, the used biotests or biochemical assays should be fast, cheap and highly automated. The safety factors used in toxicologically motivated legislation are a special issue in this context. By definition, the use of a safety factor should guarantee that e.g., a food product is safe for consumption. A sample near the legal concentration limit is expected to cause essentially no effect at all. *This means that effect-related analysis is usually not able to detect toxic compounds at these limits*. Only systems with some enrichment or other “enhancement” might detect toxic risks in such real samples, except for very high concentrations of the toxicant, which are obviously rare events.

## 12. Future Prospects

Although some of the seminal papers in this field have been published decades ago, a huge field of novel applications waits for exploration and practical application. Effect-related analysis is a highly interdisciplinary endeavor, which might be put into practice in labs with some expertise in biotechnology, biochemistry, toxicology and instrumental analysis. Novel applications might be identified e.g., in the field of doping screening, veterinary analysis, identification of active ingredients in phytotherapy, Registration, Evaluation, Authorisation and Restriction of Chemicals (REACH), monitoring of chemical and biological weapons and as a complement to a more general non-target analysis [133,145,167–171] or non-target screening [172,173]. This might finally lead to a true paradigm shift in analytical chemistry.

## Figures and Tables

**Table 1.** Overview of the nomenclature and their use in the literature.

**Designation**	**Records in ISI Web of Science** *	**References** **
bioassay-guided fractionation	1,515	[7,8]
gas chromatography-olfactometry	632	[9–13]
activity-guided fractionation	400	[14]
bioassay-directed fractionation	352	[15,16]
bioactivity-guided fractionation	341	[17]
biochemical detection	260	[18–23]
toxicity-identification evaluation	202	[24,25]
GC with electroantennographic detection	124	[26,27]
activity-directed fractionation	93	[28]
bio(-)guided fractionation	89	[29,30]
effect-directed analysis	80	[31–34]
bioactivity screening	38	[35,36]
bioautographic screening	32	[37,38]
bioassay-directed chemical analysis	19	[39,40]
toxicity-based fractionation	16	[41–43]
bioaffinity detection	8	[44]
bioassay-directed identification	7	[45–49]
bioresponse-linked instrumental analysis	6	[50–52]
toxicity-directed fractionation	5	[53–55]
bioaffinity screening	3	[56]
toxicity-directed analysis	3	[57]
bioeffect(s)-related analysis	2	[58]
bioaffinity profiling	2	[59,60]
Effect(s)-directed investigations	2	[61,62]

* Number of publications;

** Example(s).
